# Photocatalytic Dye and Cr^(VI)^ Degradation Using a Metal-Free Polymeric g-C_3_N_4_ Synthesized from Solvent-Treated Urea

**DOI:** 10.3390/polym11010182

**Published:** 2019-01-21

**Authors:** Chechia Hu, Yi-Ching Chu, Yan-Ru Lin, Hung-Chun Yang, Ke-Hsuan Wang

**Affiliations:** 1 Department of Chemical Engineering, R&D Center for Membrane Technology and Research Center for Circular Economy, Chung Yuan Christian University, Chungli Dist., Taoyuan 32023, Taiwan; vivianbobo29@gmail.com (Y.-C.C.); ken90433ken@gmail.com (Y.-R.L.); hahaha06050605@gmail.com (H.-C.Y.); 2 Department of Industrial Chemistry, Tokyo University of Science, Shinjuku-ku, Tokyo 1620826, Japan; wang@ci.kagu.tus.ac.jp

**Keywords:** Cr^(VI)^ reduction, g-C_3_N_4_, photocatalytic degradation, solvent-treated urea

## Abstract

The development of visible-light-driven polymeric g-C_3_N_4_ is in response to an emerging demand for the photocatalytic dye degradation and reduction of hexavalent chromium ions. We report the synthesis of g-C_3_N_4_ from urea treated with various solvents such as methanol, ethanol, and ethylene glycol. The samples were characterized and the Williamson–Hall method was applied to investigate the lattice strain of the samples. The activity of the samples was evaluated by observing the degradation of methyl orange and K_2_Cr_2_O_7_ solution under light irradiation. Photocatalytic reaction kinetics were determined as pseudo-first-order and zero-order for the degradation of methyl orange and reduction of hexavalent chromium, respectively. Due to the inhibited charge separation resulting from the small lattice strain, reduced crystal imperfection, and sheet-like structure, g-C_3_N_4_ obtained from ethanol-treated urea exhibited the highest activity among the evaluated samples.

## 1. Introduction

Aqueous dye solutions of methyl blue, methyl orange, rhodamine B, Congo red, etc., are commonly adopted in the textile, paper, leather, pharmaceutical, and cosmetic industries, and often cause serious problems when discharged into water resources [1,2,3]. In addition, effluent containing hexavalent chromium (Cr^(VI)^) is a critical pollutant because it is highly toxic to the human body and deleterious to the environment [4,5,6]. Therefore, the removal of Cr^(VI)^ and degradation of dye molecules in the effluent of the textile industry through photocatalytic processes to achieve a sustainable, green, and cost-effective approach is an urgent demand. Photocatalytic materials could activate under UV and/or visible light to generate active species, such as photoexcited electrons, holes, hydroxyl radicals, and superoxide radicals, to trigger the degradation [7,8,9] of dyes and reduction of metal ions (e.g., Cr^(VI)^, As^(V)^) [10,11]. Although many materials have been investigated and used as photocatalysts, visible-light-active photocatalysts that can effectively and efficiently utilize solar light are still limited [12,13,14].

In the past few decades, a metal-free polymeric material, carbon nitride (g-C_3_N_4_) with 2-D graphitic features, has been reported to serve as an efficient photocatalyst and catalyst for water splitting, CO_2_ reduction, dye degradation, and CO_2_ conversion [15,16,17,18,19]. Researchers worldwide have focused on g-C_3_N_4_ because of its high thermal and chemical stability, visible light activity, unique electronic properties, and ease of synthesis. The high thermal and chemical stability of g-C_3_N_4_ is mainly attributed to its π-conjugated stacked structure composed of repeated tri-s-triazine units and the van der Waal forces between the layered sheets [20]. Generally, graphitic-like C_3_N_4_ can be prepared via thermal condensation using various kinds of raw materials consisting of R–C–NH_2_ units, including melamine, cyanamide, dicyandiamide, thiourea, urea, and their mixtures [21]. Among these materials, melamine and urea have been proven as highly active precursors for the synthesis of g-C_3_N_4_ due to their nitrogen-rich character. Moreover, the molecular structure of melamine can be viewed as a trimer of cyanamide, which is similar to the structure of g-C_3_N_4_. Investigations on cyanamide-based materials and their synthesis date back to the 1940s, as reported by Lucas [22]. Different intermediates derived from cyameluric acid, including melam, melem, and melon, were explored. The –NH_3_ groups of the melon intermediate (with abundant amino groups) are easily eliminated produce graphitic carbon nitride with tri-s-triazine units [23]. However, cyanamide and dicyandiamide are not suitable for further laboratory- and industrial-scale production owing to their high toxicity and expense. Interestingly, urea with the chemical formula (NH_2_)_2_CO has been used in the large-scale synthesis of porous g-C_3_N_4_, which exhibits high yield, photocatalytic activity, and high stability [24]. To further increase the surface area and shorten the electron conductive path, physical design by nanostructural engineering is extensively utilized to alternatively tune the porosity, size, shape, and morphology of g-C_3_N_4_ photocatalysts. A series of conventional exfoliation methods including ultrasonication, liquid/acid treatment, and thermal oxidation etching, have been employed [25,26,27]. Post-thermal exfoliation of g-C_3_N_4_ derived from urea generated a nanosheet-like structure, leading to an extended visible light response and increased specific surface area, and hence outstanding photocatalytic performance. Apart from the surface area and visible light response, urea-derived g-C_3_N_4_ undergoes fast injection of charge carriers into localized states and inhibits radiative emission, leading to improved activity [28]. However, the aforementioned methods are mainly conducted after the formation of g-C_3_N_4_, namely post-treatment, which may induce defect formation and hence limit the photocatalytic activity. To effectively prepare polymeric g-C_3_N_4_ with reduced crystal imperfection and high specific surface area to improve the photocatalytic activity, the precursors should be carefully determined from the viewpoint of physical and chemical aspects [29]. Various solvents with different polarities could possibly lead to different internal forces between solvent molecules and the precursor, thus affecting the crystallization of polymeric g-C_3_N_4_. To the best of our knowledge, such a solvent treatment of the precursor to prepare polymeric g-C_3_N_4_ has not yet been reported.

In this study, urea (as a raw material) is treated with different solvents, including methanol, ethanol, and ethylene glycol, prior to the thermal condensation process in an attempt to clarify the influence of pre-treatment of the precursors on the synthesis of g-C_3_N_4_. The crystalline structure, surface functional groups, morphology, photoluminescence, and photocatalytic degradation of methyl orange (MO) and reduction of Cr^(VI)^ are investigated in detail. Conventional melamine-synthesized g-C_3_N_4_ is also evaluated for comparison. The present results suggest that urea-derived g-C_3_N_4_ exhibits high photocatalytic activity for the degradation of MO and reduction of Cr^(VI)^ ions, and that pretreatment of the precursor using ethanol is an effective technique for reducing the defect density and hence improving the photocatalytic activity of g-C_3_N_4_.

## 2. Experimental Procedures

Graphitic carbon nitride (g-C_3_N_4_) was synthesized by a conventional thermal condensation method using urea as a precursor. Before the synthesis process, the precursor (urea) was solvothermally treated using methanol, ethanol, or ethylene glycol at 75 °C for 12 h, where the respective samples were termed Me-urea, Et-urea, and Eg-urea. In the synthesis of g-C_3_N_4_, a certain amount of solvent-treated urea was placed into a furnace and heated at 550 °C for 6 h at a rate of 2 °C min^−1^. After heat treatment, the products were washed with deionized water several times and dried overnight at 60 °C. The products were designated based on the precursors as MeCN, EtCN, and EgCN, respectively. For comparison, untreated urea or melamine was used as a raw material for synthesizing g-C_3_N_4_ using an otherwise identical procedure, and these samples were denoted uCN and mCN, where u and m indicated urea and melamine, respectively. Regardless of the solvents used in the pretreatment, the yields of g-C_3_N_4_ (EtCN, MeCN, EgCN, uCN, and mCN) using solvent-treated urea and melamine were estimated to be approximately 12% and 45%, respectively.

X-ray diffraction (XRD) patterns were collected at 2*θ* angles of 5–60° at 40 kV, 40 mA, and a scan rate of 4° min^−1^ using a diffractometer (XRD; D8 advance, Bruker, Billerica, Massachusetts, USA) to evaluate the crystalline structure of g-C_3_N_4_. The surface functional groups of the products were analyzed using Fourier transform infrared spectroscopy (FTIR; Tensor 27, Bruker, Billerica, Massachusetts, USA). Scanning electron microscopy (SEM; S-4800N, Hitachi, Tokyo, Japan) was used to investigate the surface morphology and microstructures of the g-C_3_N_4_ specimens. The specific surface areas of mCN and uCN were determined by BET equation through N_2_ adsorption–desorption isotherm using an adsorption apparatus (Micromeritics ASAP 2020, Georgia, USA). The optical properties, including the UV-Vis absorption (UV-vis) and photoluminescence (PL), were investigated at room temperature using a UV-Vis-NIR spectrometer (U-3900, Hitachi, Tokyo, Japan) equipped with an integration sphere and a Spex Fluorolog-3 spectrofluorometer (Horiba, Kyoto, Japan) equipped with a 450 W xenon light source and double excitation monochromators. The photoluminescence emission was measured using a Photonics R928-type photomultiplier (Hamamatsu, Shizuoka Pref., Japan) placed perpendicular to the excitation beam.

Photocatalytic degradation reactions were conducted by suspending 0.25 g of photocatalyst powder in 230 mL of a methyl orange solution (MO, 2.5 × 10^−5^ M) or K_2_Cr_2_O_7_ solution (50 ppm) in a reactor cell made of Pyrex glass with continuous stirring using a magnetic stirrer. The light source was a 300 W xenon lamp. The distance between the light source and the reactor was approximately 10 cm. The degradation rate was measured from UV-vis spectra at 464 nm and 372 nm for MO degradation and Cr^(VI)^ reduction, respectively.

## 3. Results and Discussion

**Figure 1:**
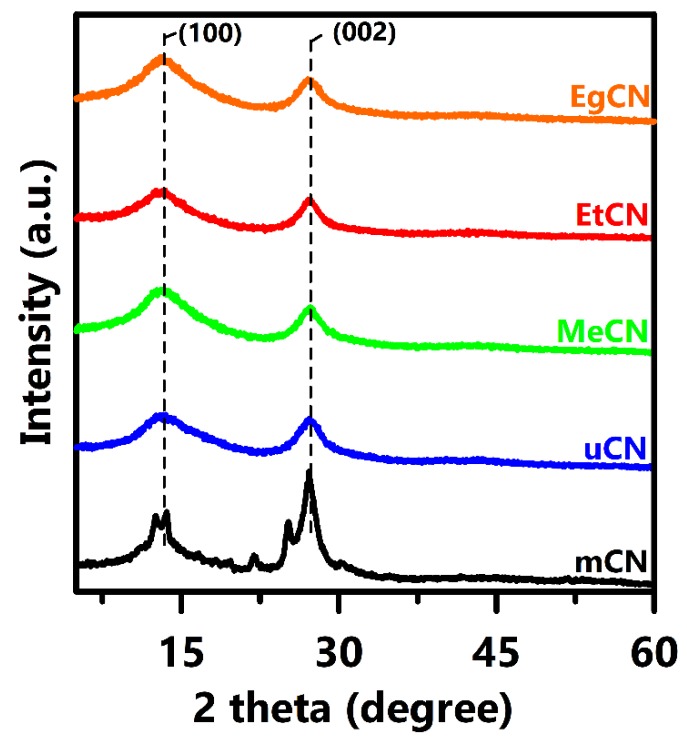
XRD patterns of mCN, uCN, MeCN, EtCN, and EgCN samples.

XRD was used to probe the crystalline structure of the g-C_3_N_4_ samples synthesized using solvent-treated urea (Figure 1). Prominent diffraction peaks were observed at 2*θ* values of 13.1 and 27.1, corresponding to the (100) and (002) planes, respectively. The interlayer spacing of the (100) plane was estimated to be 0.65−0.68 nm, revealing the distance between each tri-s-triazine unit along the (100) direction, whereas the stacking distance of the conjugated system in the (002) direction was approximately 0.32−0.33 nm. These results suggest that the interlayer distance changed slightly. Apart from the characteristic peaks of g-C_3_N_4_, several peaks can be observed in the mCN sample, showing the presence of the impurities. The Williamson–Hall (W–H) method [30] was used to evaluate the lattice strain of the samples (Figure S1). The W–H method simultaneously considers the lattice strain and the crystallite size of the sample based on Equation (1), otherwise termed the uniform deformation model (UDM):

βhklcosθ = (Kλ/D_W–H_) + 4εsinθ (1)

where *β_hkl_* is the peak width at half-maximum intensity of the (*hkl*) plane, *D_W–H_* accounts for the crystallite size, K is a constant, *λ* is the wavelength of the incident X-ray (1.5418 Å for CuKα radiation), and *ε* represents the lattice strain from crystal imperfection and distortion. Therefore, *ε* and *D_W–H_* can be estimated from the slope and intercept by plotting the term *β_hkl_*cos*θ* with respect to 4sin*θ* (Figure S1). The estimated values of *ε* and *D_W–H_* are summarized in Table 1, showing that EtCN exhibited the lowest *ε* among these samples. The positive *ε* values suggest that the lattice expanded slightly along the crystallographic axes, indicating the isotropic nature and that the strain is uniformly distributed within the crystal [31]. This implies the EtCN has the smallest lattice strain and reduced crystal imperfection, which is advantageous for further photocatalytic measurement. The crystallite size could also be obtained from the most-commonly used Scherrer equation (2):

βhklcosθ = (Kλ/Ds)(2)

where *D_s_* is the crystallite size; these data are consistent with the values obtained from the W–H method, suggesting high reliability of the calculation (Table 1).

**Figure 2:**
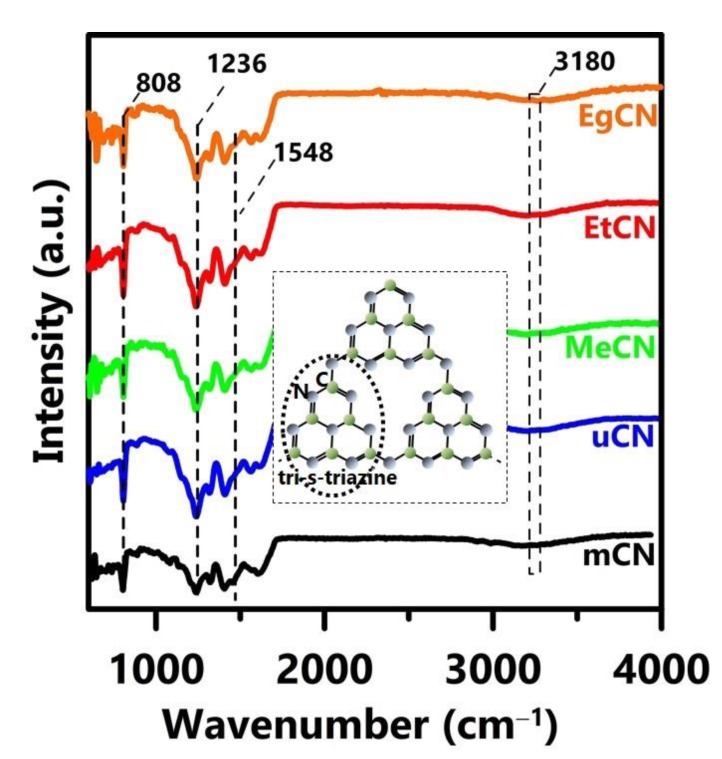
FTIR spectra of mCN, uCN, MeCN, EtCN, and EgCN samples. Inset shows the proposed molecular structure of g-C_3_N_4_.

The surface functional groups of the specimens were explored using FTIR spectroscopy (Figure S2 and Figure 2). Figure S2 shows the data for the solvent-treated precursors in the range of 600–4000 cm^−1^, displaying strong bands at 790, 1155, 1468, 1605, 1627, and 1687 cm^−1^, which can be assigned to the C=O wagging, NH_2_ rocking, C−N stretching, O−alkyl derivatives of iso-urea, N−H bending, and C=O stretching modes, respectively. The bands at 3310 and 3420 cm^−1^ are attributable to N−H in-phase and out-of-phase stretching vibrations within the (NH_2_)_2_CO structure. These results demonstrate that the chemical formula and composition of solvent-treated urea did not change significantly after the pretreatment processes. Figure 2 shows several prominent bands of the g-C_3_N_4_ samples at 808, 1236, 1548, and 3180 cm^−1^, which correspond to the characteristic breathing mode of the triazine units, C–NH–C units of melem, C=N (sp^2^) stretching vibration modes, and stretching modes of the secondary and primary amines, where intermolecular hydrogen-bonding interactions were active [32,33]. This implies the typical graphitic features of g-C_3_N_4_ and its repeated aromatic tri-s-triazine units are present, as shown in the inset of Figure 2.

**Figure 3:**
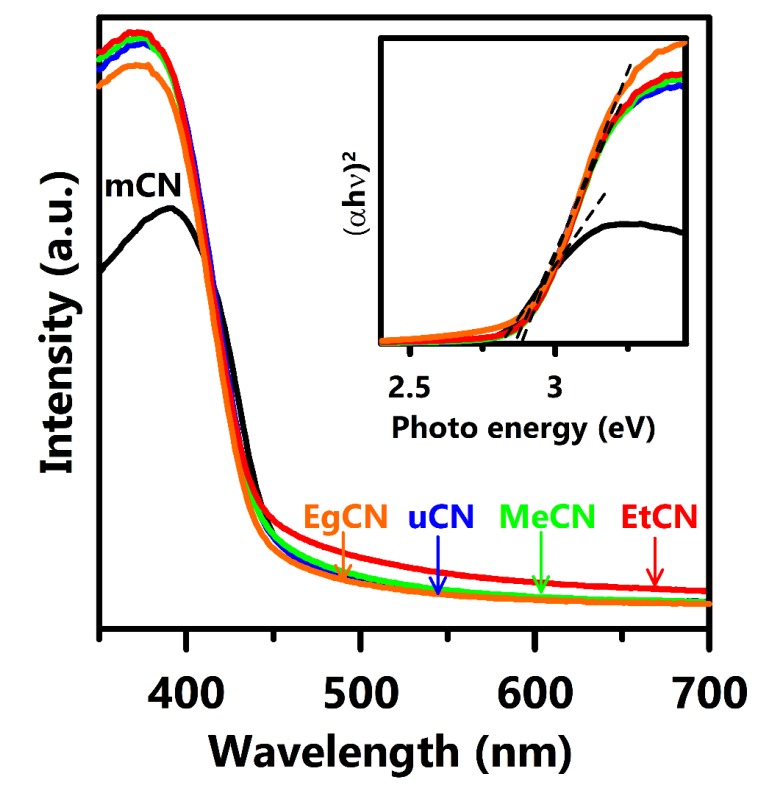
UV-vis spectra of mCN, uCN, MeCN, EtCN, and EgCN samples. Inset shows the Tauc plot of these samples.

UV-vis spectra were acquired to probe the optical properties of the g-C_3_N_4_ samples (Figure 3). The absorption band edges were located at 450 nm in the spectra, revealing that electrons in the g-C_3_N_4_ units could be excited by visible light. The absorption threshold of the urea-synthesized g-C_3_N_4_ samples did not shift towards longer wavelengths compared to that of mCN. The band-gap energies of these samples were evaluated to be ca. 2.72–2.8 eV using the Tauc plot, as shown in the inset, which is consistent with the value reported in previous literature [32]. This result strongly suggests that the optical properties do not contribute to the enhancement of the photocatalytic activity of the samples.

**Figure 4:**
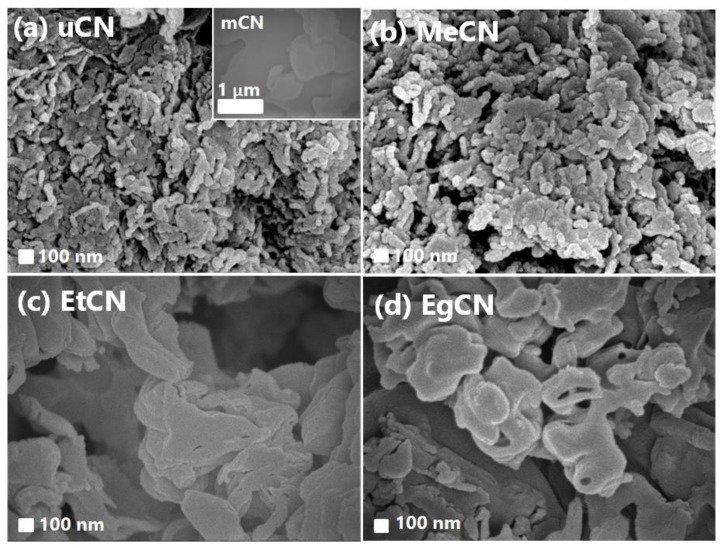
SEM images of (**a**) uCN, (**b**) MeCN, (**c**) EtCN, and (**d**) EgCN samples. Inset of (**a**) shows an image of mCN.

The surface morphologies of mCN, uCN, EtCN, MeCN, and EgCN are displayed in Figure 4. The inset in Figure 4a shows an image of mCN, showing an aggregated structure with an irregular shape. Without solvent treatment, uCN had an irregular rod-like structure with a particle length of 300–500 nm and width of 50–80 nm (Figure 4a). The morphology of MeCN synthesized from methanol-treated urea was similar to that of uCN. On the other hand, EtCN obtained from urea after ethanol treatment exhibited an irregular sheet-like structure, which may provide a large contact area between each layer, as well as a shorter distance for electron conductance. This could effectively facilitate charge migration and hence enhance the photocatalytic activity, similar to a previous report [28]. As shown in Figure 4d, EgCN prepared from ethylene glycol-treated urea had larger particle sizes than the mCN, uCN, MeCN, and EtCN samples. The BET specific surface areas of mCN and uCN were 8.8 and 115.1 m^2^/g, respectively. The increased specific surface area of uCN is consistent with the above SEM observations. The structural changes could be partially attributed to solvent polarity, where the corresponding values for methanol, ethanol, and ethylene glycol were 0.762, 0.654, and 0.790, respectively. The use of a more polar solvent in the solvothermal process during the pretreatment of urea may lead to fast condensation and crystallization [29]. As a result, in the further thermal condensation process for the synthesis of g-C_3_N_4_, ethanol-treated urea with the slowest crystallization and reduced crystal imperfection (Table 1) exhibited a sheet-like structure, whereas the samples prepared from ethylene glycol- and methanol-treated urea had an irregular and rod-like structure.

**Figure 5:**
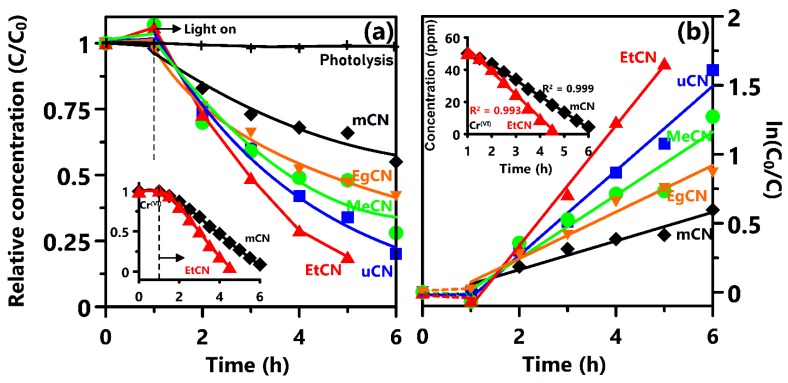
(**a**) Photocatalytic degradation of MO aqueous solution (2.5 × 10^−5^ M) using mCN, uCN, MeCN, EtCN, and EgCN samples under irradiation from a Xe lamp, and (**b**) the pseudo-first-order reaction kinetics plot for these samples. Insets of (**a**) and (**b**) show the photocatalytic reduction of Cr^(VI)^ using the mCN and EtCN samples, and their pseudo-zero-order reaction kinetics, respectively.

The photocatalytic activity of the g-C_3_N_4_ samples was evaluated using MO and Cr^(VI)^ as target compounds under visible light irradiation. Figure 5a shows the changes in the concentration of MO as a function of the irradiation time, demonstrating that self-degradation and photolysis were negligible in the initial process. Notably, EtCN exhibited the highest degradation rate, whereas that of mCN was the lowest. The kinetics of photocatalytic degradation were studied, as shown in Figure 5b. The experimental data were well fitted to pseudo-first-order kinetics with a simplified Langmuir–Hinshelwood model with the assumption that *C*_0_ was relatively small based on the equation ln(*C*_0_/*C*) = *kt*, where *C*_0_ and *C* are the concentration at time *t_0_* and *t* and *k* is the rate constant. EtCN had a rate constant of 0.436 h^−1^, which is almost 4.2 and 1.42 times higher than that of mCN and uCN, respectively. In the reusability test for the photocatalytic MO degradation, EtCN exhibited a high stability and maintained over 90% efficiency during three cycles of MO degradation (Figure S3). The photocatalytic MO degradation mechanism using g-C_3_N_4_ was reported by Tong et al. in 2015 [34]. The main reactive species responsible for the MO degradation are •O_2_^–^ and •OH, while h^+^ only affected the reaction slightly. The inset in Figure 5a shows the photocatalytic reduction of K_2_Cr_2_O_7_ solution (50 ppm) using the EtCN and mCN samples, demonstrating that EtCN enables the reduction of Cr^(VI)^ within 5 h of irradiation. On the other hand, the zero-order kinetics equation (*C* = *C*_0_−*k’t*) was adopted to express the photoreduction of Cr^(VI)^, showing the linear relationship with a regression factor larger than 0.99 (inset of Figure 5b). The observed pseudo-zero-order rate constant *k’* was 14 and 9 (ppm h^−1^) for EtCN and mCN, respectively. This indicates that the concentration of Cr^(VI)^ does not affect the reaction rate due to the full coverage of the catalyst surface by the substance and the degraded species. Cr^(VI)^ ions interacted with the photoexcited electrons from g-C_3_N_4_ surface sites for the reduction to harmless Cr^(III)^ ions with low valent states [35]. The detailed Cr^(VI)^ reduction mechanism, including the implied and excluded molecules and ions involved in the reduction and the interaction between EtCN and these species, will be discussed in the future. Table 2 summarizes the photocatalytic MO degradation and Cr^(VI)^ reduction using various g-C_3_N_4_ samples [36,37,38,39,40,41,42,43,44,45,46,47,48,49,50,51,52,53,54,55], again confirming that ethanol pretreatment is an efficient way to improve the photocatalytic activity of the evaluated species under visible light irradiation for environmental remediation.

**Figure 6:**
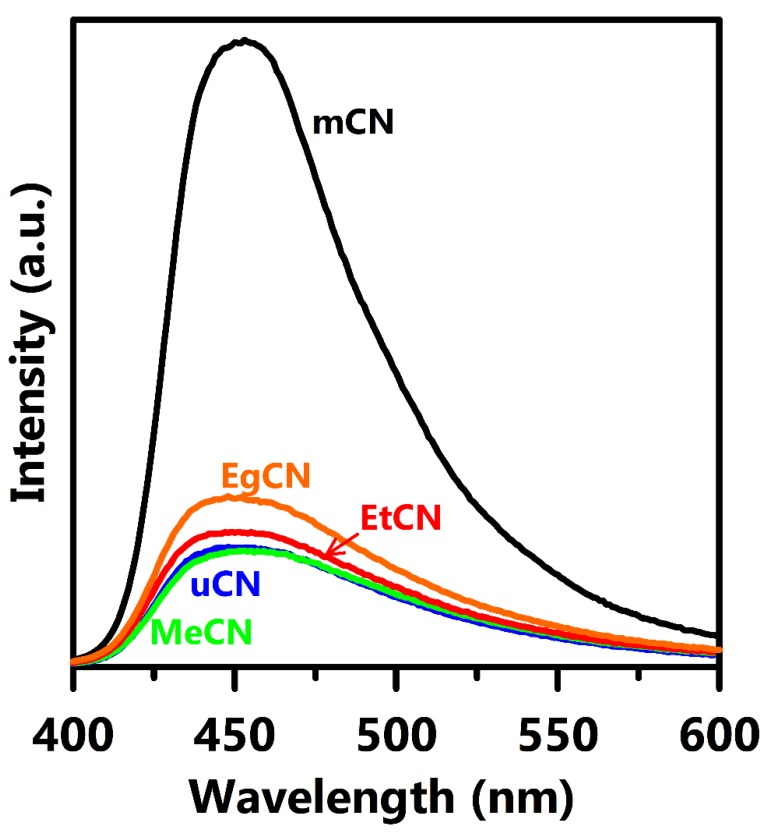
Photoluminescence spectra of mCN, uCN, MeCN, EtCN, and EgCN samples excited at wavelength of 360 nm at room temperature.

To examine the photoluminescence emission and the charge recombination, the PL spectra were acquired at a wavelength of 360 nm (Figure 6). The g-C_3_N_4_ samples all exhibited a strong emission band at approximately 460 nm, which is attributed to intrinsic electron-hole recombination between the conduction and valence band. The intensity of this band for the mCN sample was much higher than that of the EgCN, EtCN, uCN, and MeCN samples, implying that charge recombination can be greatly inhibited through proper pretreatment of the raw material. This can be ascribed to the lower amount of lattice strain (Figure S1, Table 1) and the single g-C_3_N_4_ phase (Figure 1) of the samples employing solvent-treated urea as the precursor. The reduced intensity of the PL emission in CN synthesized from solvent-treated urea indicates effective charge separation, and hence efficient photocatalytic activity, which is similar to the data presented in previous literature.

**Figure 7:**
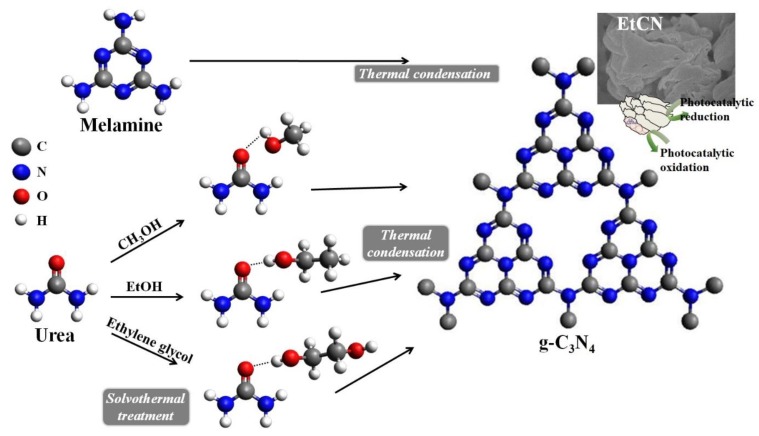
Proposed mechanism for synthesis of g-C_3_N_4_ using melamine or solvent-treated urea as raw materials.

A possible mechanism of formation of g-C_3_N_4_ obtained from solvent-treated urea is presented in Figure 7. Thermal condensation of melamine (C_3_N_3_(NH_2_)_3_) resulted in an elimination of the terminal −NH_2_ groups, followed by polymerization of these repeating units for the g-C_3_N_4_ structure with tri-s-triazine rings. When solvent-treated urea was used as the raw material, these solvent molecules tended to interconnect with the terminal −C=O group of urea via inter-hydrogen bonds during the solvothermal treatment. During the further thermal condensation process, heating of these solvent-treated urea molecules yielded graphitic carbon nitride at the desired temperature. Since ethanol has the lowest polarity among these solvents (methanol, ethanol, and ethylene glycol), the internal force between solvent molecules and the urea should be low, resulting in slow crystallization [29] and reduced crystal imperfection. As a result, the ethanol-treated urea-derived g-C_3_N_4_ sample exhibited fast charge carrier injection to localized states [21], leading to high photocatalytic performance.

## 4. Conclusions

In conclusion, the synthesis of polymeric g-C_3_N_4_ from solvent-treated urea was demonstrated and photocatalytic analysis was performed. g-C_3_N_4_ obtained from ethanol-treated urea exhibited the highest photocatalytic activity in the degradation of MO and the reduction of Cr^(VI)^ under irradiation with a Xe lamp. The photocatalytic reactions for MO degradation and Cr^(VI)^ reduction follow pseudo-first- and pseudo-zero-order kinetics, respectively. The rate constants of the EtCN sample were estimated to be 0.436 h^−1^ and 14 ppm h^−1^ for photocatalytic MO and Cr^(VI)^ removal, respectively. The improved photocatalytic activity of EtCN can be attributed to its inhibited charge recombination emission, reduced crystal imperfections, and sheet-like structure. Current research focuses on the characterization and synthesis of g-C_3_N_4_ obtained from solvent-treated urea and its effect on morphology, crystalline structure, and photocatalytic applications. The various solvents with different polarities could affect the internal force between the solvent molecules and the precursor, resulting in the different structural features of the g-C_3_N_4_ sample. The present data strongly suggest that the pretreatment of the precursor is a rapid and simple method to alternatively prepare an effective g-C_3_N_4_ as a photocatalyst for environmental remediation.

## Figures and Tables

**Table 1:** Lattice strain (*ε*) and crystallite size (*D_W–H_* and *D_s_*) obtained from Williamson–Hall and Scherrer methods for uCN, MeCN, EtCN, and EgCN samples.

Samples	Williamson–Hall Method		Scherrer Method	
	*ε*	*D_W–H_* (nm)	*D_s(100)_* (nm)	*D_s(002)_* (nm)
uCN	0.0405	2.494	1.52	3.49
MeCN	0.0414	2.478	1.71	3.82
EtCN	0.0403	2.453	2.12	3.74
EgCN	0.0413	2.476	1.72	3.84

**Table 2:** Reported reaction conditions, including light source, original concentration of MO or Cr^(VI)^ solution, and photocatalytic performance using various polymeric g-C_3_N_4_ samples.

**Samples**	**Light Source**	**Rate Constant, k (h^−1^)**	**Amounts of Catalyst (g)**	**MO Concentration (ppm)**	**Year**	**Ref.**
g-C_3_N_4/_BiOCl	300 W Xe lamp with an optical filter (>420 nm)	0.0858	0.01	10	2017	[36]
g-C_3_N_4_/POM (Polyoxometalates)	300 W Xe lamp with an optical filter (>420 nm)	2.28	0.05	20	2017	[37]
β-C_3_N_4_/CuO	300 W UV lamp	0.0311	0.005	10	2017	[38]
g-C_3_N_4_/Bi_2_S_3_	350 W Xe lamp	0.2874	0.05	10	2018	[39]
g-C_3_N_4_	500 W Xe lamp with an optical filter (>420 nm)	0.1266	0.25	10	2018	[40]
g-C_3_N_4_/CdS	300 W Xe lamp with an optical filter (>420 nm)	0.306	0.1	10	2018	[41]
g-C_3_N_4_/YVO_4_	300 W Xe lamp	0.6786	0.02	20	2018	[42]
g-C_3_N_4_/Bi_2_WO_6_	500 W Xe lamp with an optical filter (>420 nm)	0.5994	0.05	5	2018	[43]
g-C_3_N_4_/Ag/MoS_2_	350 W Xe lamp with an optical filter (>420 nm)	0.78	0.05	10	2018	[44]
g-C_3_N_4_/CuCo_2_O_4_	150 W Xe lamp	0.138	0.03	10	2018	[45]
**Samples**	**Light source**	**Rate Constant, *k* (min^−1^)**	**Amounts of Catalyst (g)**	**Concentration of K_2_Cr_2_O_7_ (ppm)**	**Year**	**Ref.**
acid-treated g-C_3_N_4_	Visible light with an optical filter (>420 nm)	/	0.3	50	2015	[46]
g-C_3_N_4_/α-Fe_2_O_3_	300 W Xe lamp	/	0.1	10	2015	[47]
g-C_3_N_4_	Visible light with an optical filter (>420 nm)	0.0025	0.3	50	2016	[48]
g-C_3_N_4_/SnS_2_	Visible light with an optical filter (>420 nm)	0.0109	0.3	50	2017	[49]
g-C_3_N_4_/MIL53(Fe)	500 W Xe lamp with an optical filter (760 > λ > 420 nm)	0.004	0.02	10	2017	[50]
S-doped g-C_3_N_4_	300 W Xe lamp with an optical filter (>400 nm)	0.0036	0.01	5	2017	[51]
g-C_3_N_4_/SnS_2_/SnO_2_	300 W Xe lamp	0.001	0.05	20	2017	[52]
g-C_3_N_4_	30 W white LED	/	0.05	2.94	2017	[53]
g-C_3_N_4_ nanosheet	300 W Xe lamp with an optical filter (>400 nm)	/	0.05	20	2017	[54]
g-C_3_N_4_/Au	300 W Xe lamp with an optical filter (>400 nm)	/	0.02	20	2018	[55]
g-C_3_N_4_ obtained from ethanol-treated urea	350 W Xe lamp (>400 nm)	MO: 0.436 h^−1^ Cr^(VI)^: 14 ppm h^−1^	0.25	MO: 2.5 × 10^−5^ M (8.2 ppm) K_2_Cr_2_O_7_ solution: 50 ppm	This work (2018)	

## Supplementary Materials

The following are available online at http://www.mdpi.com/2073-4360/11/1/182/s1: Figure S1, Williamson–Hall plot for uCN, MeCN, EtCN, and EgCN samples, Figure S2, FTIR spectra of urea treated with various solvents including methanol (Me-urea), ethanol (Et-urea), and ethylene glycol (Eg-urea), and Figure S3, reusability of the EtCN photocatalyst for the photocatalytic MO degradation during three cycles. The degradation efficiency is calculated based on the rate constant (*k*).

## Abbreviations

*C*_0_	dye concentration in time *t*_0_ (M)_._
*C*	dye concentration in time *t* (M).
*D_s_*	crystallite size obtained from Scherrer method (nm).
*D_W-H_*	crystallite size obtained from Williamson-Hall method (nm).
*k*	first-order reaction rate constant (h^−1^).
*k*’	zero-order reaction rate constant (ppm h^−1^).
*K*	shape factor (/)
*t*_0_	initial irradiation time (h)
*t*	irradiation time (h)
*β_hkl_*	full width at half maximum at (*hkl*) plane.
*ε*	lattice strain obtained from the Williamson-Hall method.
*λ*	wavelength of incident X-ray (1.5406 Å)
*θ*	XRD diffraction angle (degree).
