# Supplementary material for: Photocatalytic Dye and Cr(VI) Degradation Using a Metal-Free Polymeric g-C3N4 Synthesized from Solvent-Treated Urea
Source: Polymers (Basel). 2019 Jan 21;11(1):182. doi: 10.3390/polym11010182 (PMC6401860; doi:10.3390/polym11010182)
Supplement: Supplementary file 1 [file polymers-11-00182-s001.pdf]

Photocatalytic dye and Cr(VI) degradation using a metal-free polymeric g-C_3N_4 synthesized from solvent-treated urea

Chechia Hu[a*], Yi-Ching Chu[a], Yan-Ru Lin[a], Hung-Chun Yang[a], Ke-Hsuan Wang[b]

[a] Department of Chemical Engineering and R&D Center for Membrane Technology, Chung Yuan Christian University, Chungli Dist., Taoyuan City, Taiwan 32023

[b] Department of Industrial Chemistry, Tokyo University of Science, Shinjuku-ku, Tokyo, Japan

* To whom correspondence should be addressed. E-mail: chechiahu@cycu.edu.tw

Tel: +886-3-2654152 Fax: +886-3-2654199

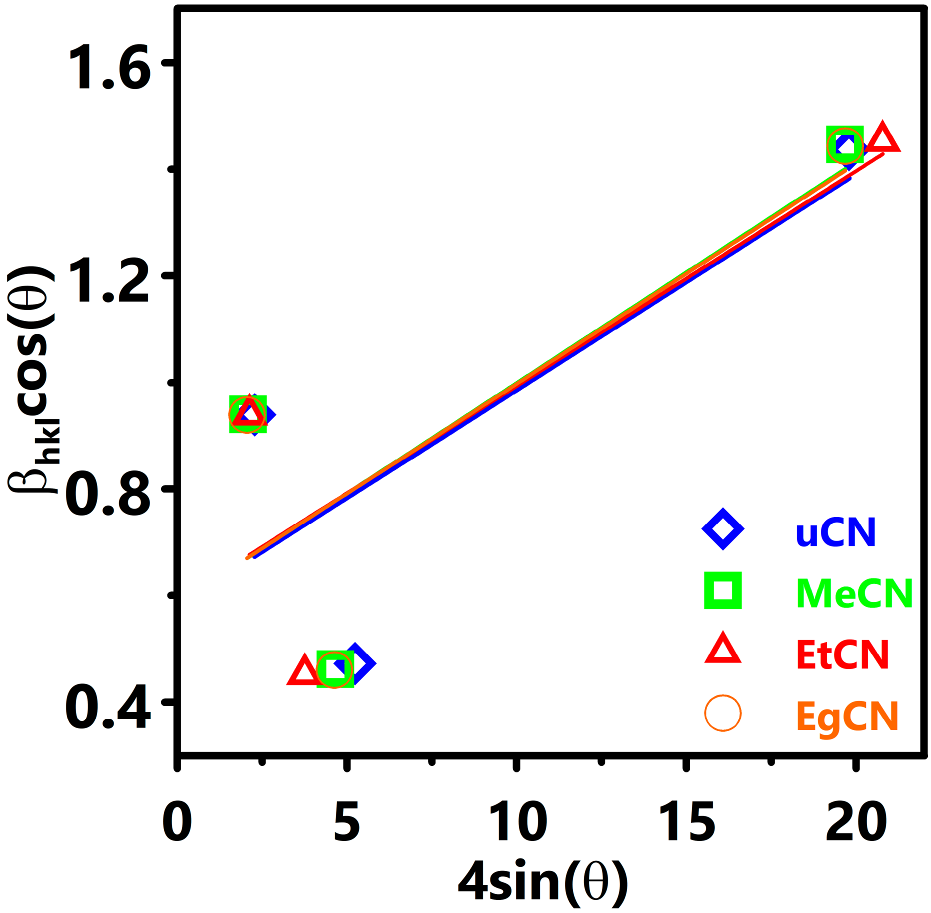

Figure S1 Williamson-Hall plot for uCN, MeCN, EtCN, and EgCN samples.

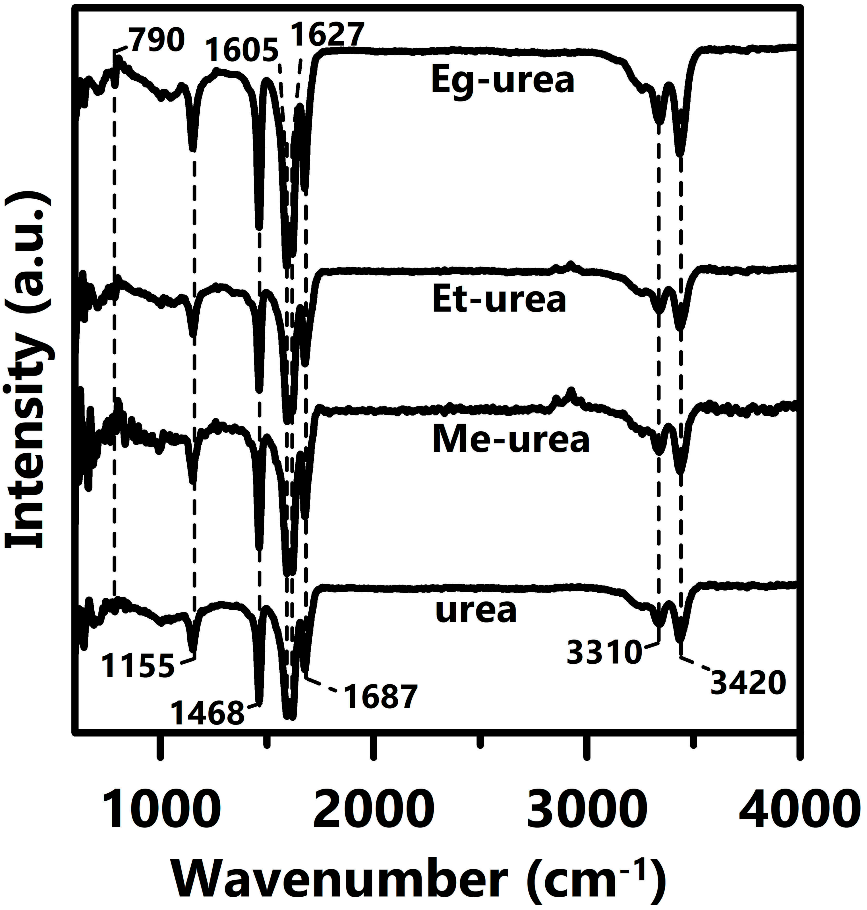

Figure S2 FTIR spectra of urea treated with various solvents including methanol (Me-urea), ethanol (Et-urea), and ethylene glycol (Eg-urea).

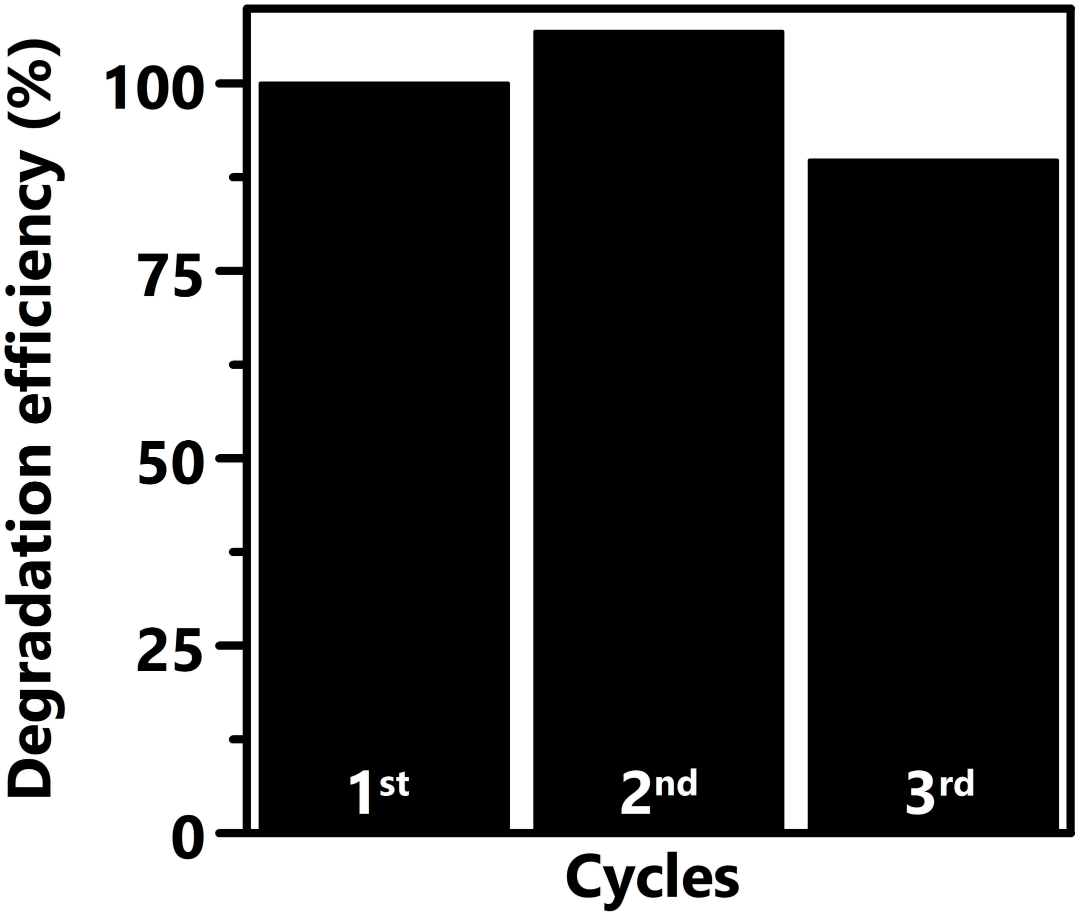

Figure S3 Reusability of the EtCN photocatalyst for the photocatalytic MO degradation during three cycles. The degradation efficiency is calculated based on the rate constant (*k*).
